# Immunomodulatory Effects of Aronia Juice Polyphenols—Results of a Randomized Placebo-Controlled Human Intervention Study and Cell Culture Experiments

**DOI:** 10.3390/antiox11071283

**Published:** 2022-06-28

**Authors:** Sonja Lackner, Tommaso Sconocchia, Tobias Ziegler, Christina Passegger, Nathalie Meier-Allard, Elke Schwarzenberger, Willibald Wonisch, Theresa Lahousen, Alexandra Kohlhammer-Dohr, Sabrina Mörkl, Martina Derler, Herbert Strobl, Sandra Johanna Holasek

**Affiliations:** 1 Division of Immunology and Pathophysiology, Otto Loewi Research Center, Medical University of Graz, 8010 Graz, Austria; sonja.lackner@medunigraz.at (S.L.); tommaso.sconocchia@medunigraz.at (T.S.); tobias.ziegler@greenbeat.at (T.Z.); christina.passegger@medunigraz.at (C.P.); nathalie.allard@medunigraz.at (N.M.-A.); el.baumgartner@medunigraz.at (E.S.); martina.derler@uni-graz.at (M.D.); herbert.strobl@medunigraz.at (H.S.); 2 Division of Haematology, Department of Internal Medicine, Medical University of Graz, 8036 Graz, Austria; 3 Juice Plus+ Science Institute, Collierville, TN 38017, USA; 4 Division of Physiological Chemistry, Otto Loewi Research Center, Medical University of Graz, 8010 Graz, Austria; willibald.wonisch@medunigraz.at; 5 Department of Psychiatry and Psychotherapeutic Medicine, Medical University of Graz, 8036 Graz, Austria; theresa.lahousen@medunigraz.at (T.L.); alexandra.kohlhammer-dohr@medunigraz.at (A.K.-D.); sabrina.moerkl@medunigraz.at (S.M.); 6 Department of Pharmacology and Toxicology, University of Graz, 8010 Graz, Austria

**Keywords:** *Aronia melanocarpa* juice, polyphenols, regulatory T cells, tolerability, oxidative stress, bioavailability, hormesis, immunomodulation

## Abstract

Dietary polyphenols, which are present in *Aronia melanocarpa,* have been associated with various beneficial effects on human health including antioxidant, antiviral, and anti-inflammatory activities. We aimed to investigate the immunomodulatory effects of aronia juice polyphenols in a randomized placebo-controlled human intervention study and cell culture experiments. A total of 40 females were asked to consume either 200 mL of aronia juice or a placebo drink for six weeks and were investigated again after a washout period of another six weeks. We observed that only half of the participants tolerated the aronia juice well (Vt) and the other half reported complaints (Vc). The placebo (P) was generally tolerated with one exception (*p* = 0.003). Plasma polyphenol levels increased significantly in Vt after the intervention (*p* = 0.024) but did neither in P nor in Vc. Regulatory T cell (Treg) frequencies remained constant in Vt and P during the intervention, whereas Tregs decreased in Vc (*p* = 0.018). In cell culture, inhibiting effects of ferulic acid (*p* = 0.0005) and catechin (*p* = 0.0393) on the differentiation of Tregs were observed as well as reduced activation of CD4-T cells in ferulic acid (*p* = 0.0072) and aronia juice (*p* = 0.0163) treated cells. Interestingly, a CD4^+^CD25^−^FoxP3^+^ cell population emerged in vitro in response to aronia juice, but not when testing individual polyphenols. In conclusion, our data strengthen possible individual hormetic effects, the importance of the food matrix for bioactivity, and the need for further investigations on possible impacts of specific physiological features such as the gut microbiota in the context of personalized nutrition.

## 1. Introduction

Polyphenols are secondary plant nutrients, and major plant dyes and are responsible for the plant’s flavor. They protect plants from herbivores and other environmental stressors and attract insects and animals beneficial for reproduction. Polyphenols occur in various fruits and vegetables, tea, coffee and cocoa, and herbs, whereas especially dark berries have a high content of polyphenols [1]. Many hundreds of polyphenols exist. According to their chemical structure, they can be allocated into two main categories: non-flavonoids (such as phenolic acid, stilbenes, coumarins, lignans, tannins) and flavonoids (such as flavonols, flavononols, favones, flavanols, flavanones, anthocyanidins, isoflavonoids) [2,3].

In recent years, plant polyphenols have been associated with various beneficial effects on human health because of their antidiabetic, cardiovascular [4], neuroprotective and antihypertensive [5] actions. Some evidence of their protective and therapeutic effects on diseases that are closely connected to immune response such as inflammatory diseases, allergic occurrences, or autoimmune diseases has been reported [6]. Further, antibacterial, antiviral, and anti-inflammatory activities of *Aronia melanocarpa* have been identified [7]. 

These positive health effects have mainly been attributed to the polyphenols’ antioxidant properties that counteract oxidative stress and chronic inflammation [8]. Moreover, the polyphenols’ involvement in immune metabolism has been the focus of research as they have been shown to contribute to immune cell proliferation and differentiation and modulate immune cell pathways [9,10]. Polyphenols inhibit proinflammatory pathways, decrease proinflammatory cytokine production and promote innate and acquired defense mechanisms [6]. Several phenolic compounds have been reported to modulate the immune system in a biphasic manner [11,12].

Within the immune system, regulatory T cells (Tregs) are key components of immunomodulation. Tregs are a subset of CD4^+^ T cells endowed with strong suppressive abilities [13]. Tregs represent a specialized subset of T cells that are key players in the regulation of self-tolerance and homeostasis. These cells are characterized by constitutive high expression of the interleukin 2 (IL-2) receptor alpha chain (CD25) and by stable expression of the Forkhead box P3 (FoxP3) transcription factor [14]. Treg cells can be divided into natural thymus-derived Tregs and induced Tregs. The former, as their name suggests, develops in the thymus and the latter develops in peripheral tissues from naïve CD4^+^ CD45RA^+^ T cells when exposed to TGF- β1 [15] and IL-2 [16]. Tregs regulate the immune response by suppressing T cell activation and proliferation through contact-dependent and independent mechanisms and this ability is of crucial importance in order to shut down inflammation and avoid an excessive inflammatory response, (i.e., following exposure to pathogenic microorganisms or dietary substances) [17]. Thus, Tregs serve as a marker of the immune response. 

The polyphenols’ impact on human health is not only related to their amount and qualities taken up by food [11] but also to the efficiency of their release from the food matrix during digestion [18]. The bioactive compounds provided by natural plant food are taken up embedded in a complex network of macromolecular structures such as dietary fibers and other nutrients. They are commonly released during digestion throughout the gastrointestinal tract; however, their absorption may also be altered by accompanying dietary components [18]. This bioaccessibility strongly influences the availability of the compounds for further metabolic processing and thus determines the bioavailability and the metabolic fate of polyphenols [2]. 

Polyphenols have a bidirectional interaction with the gut microbiota. In addition to the microbe’s enzymatic breakdown of the dietary components, polyphenols act as prebiotics. They nourish the bacteria and promote the growth of certain bacterial strains and thus modulate the microbial composition [19] which further promotes the immune system through modulation of metabolism, integrity, and immune function of enterocytes [20].

Compared to other fruits and vegetables the berry *Aronia melanocarpa* also known as chokeberry has very high polyphenol concentrations. It comprises a blend of different polyphenols, whereas proanthocyanidins, anthocyanins and the phenolic acids chlorogenic acid and neochlorogenic acids are the predominant polyphenols in aronia berries [21,22]. Proanthocyanidins are condensed tannins and are responsible for the astringent taste [23] while the high content of anthocyanins causes the dark blue color of the berries [22]. The beneficial effects of aronia polyphenols on the immune system such as the reduction in proinflammatory cytokines [7] and immunomodulation through cellular pathways [6,24] have been revealed and also the beneficial effects on other health outcomes in particular on oxidative stress, lipid metabolism, blood coagulation and hypertension [5] have been explored, leading to proposed preventive effects on cardiovascular diseases [25]. 

The great majority of publications report on the beneficial effects of aronia polyphenols on health outcomes. However, controversial data have also been published. Gajic et.al. [26] observed opposed effects on the immune response in mouse models. 

In general, clinical data to explore the effects of aronia fruits on the immune system are warranted. We investigated the effects of a six-week intervention of aronia juice polyphenols compared to a polyphenol-free placebo drink on plasma polyphenol concentrations, regulatory T cell (Treg) frequencies in blood, and oxidative stress. Additionally, we tested the effects of individual polyphenols and aronia juice on T cell activation and Treg differentiation in cell models. In total, we highlight the possible hormetic effects of aronia juice polyphenols. Thereby, the hormetic doses may be dependent on intra-individual determinants which need to be explored further and may be essential for personalized dietary approaches.

## 2. Materials and Methods

### 2.1. Study Design

**Figure 1:**
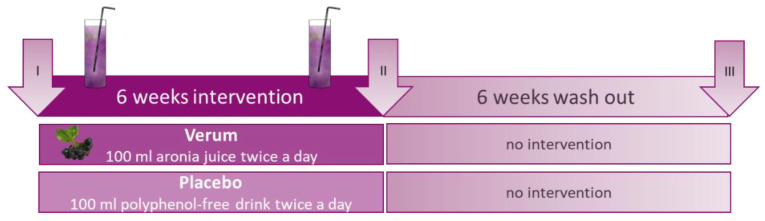
Study design: the participants were investigated at three time points at baseline (I), after the intervention period of six weeks (II), and after another six weeks of washout (III) to observe any potential persistent effects of the intervention. The participants were asked to consume 100 mL of the aronia juice or the polyphenol-free placebo drink, respectively, twice a day for six weeks.

We performed a blinded clinical placebo-controlled intervention study to test the effects of aronia juice polyphenols on plasma polyphenol levels, oxidative stress, and the frequencies of regulatory T cells (Treg) in healthy females over a period of six weeks. For another six weeks without intervention, the persistence of the effects of the polyphenols was observed (Figure 1). The study protocol included three investigation time points: at baseline (I), after the intervention (II), and after the washout period (III).

The study participants were allocated randomly to the verum group (V) who drank the aronia juice and the placebo group (P) who drank a completely polyphenol-free placebo drink. The aronia juice was derived from a local producer and the polyphenol-free placebo drink was prepared in accordance with a published recipe [27]. The nutrient profile of the placebo drink was comparable to the natural juice except for the polyphenol concentration. The average aronia juice macro- and micronutrients content was added to the placebo drink. It was colored with polyphenol-free food dyes and bottled in the same containers as the original juice. 

The group allocation was blinded to the participants. For the randomization of the group allocation, a central computerized randomization schedule (www.randomizer.at (accessed on 25 February 2019), provided by the Institute of Medical Informatics, Statistics and Documentation of the Medical University of Graz, Austria) stratified for age was applied.

The participants were asked to drink a total of 100 mL of aronia juice or the polyphenol-free placebo drink, respectively, twice a day in the morning and the evening preferentially. Thus, the total amount of juice consumed was 200 mL a day. This concentration has been chosen following previous studies testing the effects of polyphenol-rich drinks [28,29,30].

At all three study visits, blood draws were performed on overnight fasted participants, meaning the absence of food and drink intake of at least 12 h with the exception of tap water. Plasma, serum, and whole blood samples were collected for further laboratory analysis as described in Section 2.6.

### 2.2. Study Population

A total of 40 normal-weight females aged between 18–40 years were enrolled in the study. For recruitment, information on the study was spread via the local universities, libraries, sports clubs, and word of the mouth advertisement. The participants were informed about the astringent taste of the study drinks in advance to ensure that their individual preferences met the specific taste of the intervention drinks.

The study participants had to meet the following inclusion criteria for enrolment: premenopausal women aged between 18–40 years, normal body weight according to WHO criteria, tolerability to astringent taste, and the absence of the defined exclusion criteria. People with known fructose malabsorption and fructose intolerance, as well as acute diseases within the previous two months or chronic diseases or infections (including upper respiratory tract infections, fever, chronic inflammatory disorders, autoimmune disorders), a history of digestive diseases such as inflammatory bowel disease and irritable bowel syndrome, history of gastrointestinal surgery (other than appendectomy), pregnancy and period of breastfeeding, were excluded from study participation. Furthermore, antibiotic or antifungal treatment within the previous two months and daily or irregular intake of supplemental prebiotics or probiotics within the previous two months (the intake of yogurt and dairy products was permitted) were exclusion criteria. 

The participants were asked to adhere to their regular dietary and lifestyle habits during their study participation. Any smoking habits were evaluated by the Fagerström test for nicotine dependency [31].

The ethical approval for this study was obtained by the Ethics Committee of the Medical University Graz (EK: 30-009 ex 17/18) and the study was conducted in accordance with the Declaration of Helsinki. All participants gave their written informed consent and volunteered in this study. 

### 2.3. Compliance Assessment and Gastrointestinal Symptoms Questionnaire

Contact was kept regularly with the study participants via phone calls and e-mail to ensure their motivation and compliance. Any questions and queries regarding the study were discussed individually. The compliance was checked after the intervention at timepoint II by weighing the returned juice containers. To check for the tolerability of the intervention drinks the participants were asked to fill in a gastrointestinal symptoms questionnaire (adapted from [32]) and to report on side effects associated with the study drinks. 

### 2.4. Anthropometrics

Body height, body weight, and waist circumferences were measured in accordance with the International Society for the Advancement of Kinanthropometry (ISAK) standards [33] and the body mass index (BMI) was calculated according to the formula BMI = body weight [kg]/body height [m]² [34].

### 2.5. Nutritional Intake

Dietary and nutrient intake were obtained with the Vienna Food Record [35] which is based on a 4-day food record and includes Austrian-specific eating habits. The nutrient intake was calculated by the Austrian-specific nutritional software nut.s^®^ v1.32.95 (www.nutritional-software.at (accessed on 15 October 2021), dato Denkwerkzeuge, Vienna, Austria). The participants reported on their diets the four days prior to each blood draw.

### 2.6. Clinical Laboratory Parameters

Serum, plasma, and whole blood samples were taken from overnight fasted participants. The plasma and serum tubes were centrifuged for 10 min at 3000× *g* and aliquoted in 300 µL aliquots. The tubes were stored at −80 °C until analysis. Before starting the assays, the samples were thawed and centrifuged for 5 min at 10,000× *g*. Whole blood samples were analyzed within two hours after the blood was taken.

#### 2.6.1. Total Polyphenols in Plasma

For the assessment of total plasma polyphenols, the microtiter-formate Folin–Ciocalteu method was applied [36]. The polyphenols-microtitre (PPm) were determined with the PPm^®^ kit (Omnignostica GmbH, Höflein, Austria). The method is based on a colorimetric high-throughput method for the measurement of polyphenolic compounds in human samples and biological fluids. Polyphenols react with the transition metals of the Folin–Ciocalteu reagents leading to a dark blue colored complex which can be measured photometrically at 766 nm. For standardization, a serial dilution of gallic acid was used. PPm values are presented as mmol/L. PPm determination was performed in duplicates.

#### 2.6.2. Oxidative Stress Parameters

Total antioxidant capacity (TAC^®^) was measured by a colorimetric approach (Labor Diagnostic Nord, Nordhorn, Germany). This method uses 3,5,3′5′-tetra-methylbenzidine (TMB) as a colorimetric substrate to visualize the inhibition of radicals through antioxidants. Antioxidants in plasma or serum samples inhibit the effect of reactive oxygen species (ROS) on TMB that is associated with the colorimetric signal. After the incubation, the color changes from blue to yellow upon the addition of the stop solution. Total peroxides (TOC^®^) were similarly determined with a colorimetric assay (Labor Diagnostic Nord, Nordhorn, Germany). Peroxides in serum and plasma samples react with horseradish peroxidase to give a blue to a green color to the chromogenic substrate TMB. The color turns yellowish after the addition of the stop solution. Optical density for TAC and TOC was performed at a wavelength of 450 nm (reference wavelength 620 nm). A linear standard curve (up to 1 mM) was used for quantification of total peroxides [37]. TAC and TOC measurements were performed in duplicates.

To assess the relation between pro- and antioxidant conditions, the oxidative stress index (OSI) was calculated which is expressed in arbitrary units (AU) as the ratio of TOC/TAC. The OSI is comparably high in cases with a predominance of ROS and higher TOC compared to TAC indicating higher oxidative stress [38].

#### 2.6.3. Plasma Vitamin C

Vitamin C concentrations in plasma were determined with high-performance liquid chromatography (HPLC) using ClinRep^®^complete Kit (Recipe, Munich, Germany). Since Vitamin C is very sensitive to light and temperature-induced degradation, the samples were protected from light and processed immediately after the blood was taken. To 100 µL lithium-heparin plasma 100 µL precipitant, containing internal standard, was pipetted, mixed, and further incubated for 10 min at 4 °C in the dark. The samples were centrifuged at 10,000× *g* for 10 min and the supernatant was transferred into a new tube. A total of 20 µL of the supernatant were injected into the HPLC system (JASCO^®^, Vienna, Austria) and the signal measurement was performed at the UV detector with an appropriate wavelength set at 243 nm with a flow rate of 1.3 mL/min and a column temperature of 30 °C. Test solution, calibrators, and controls containing a known concentration of Vitamin C were used for system check and quantification with internal standard and one-point calibration. The prepared Vitamin C samples were measured in duplicates.

#### 2.6.4. Regulatory T Cells (Treg)

Tregs were quantified by multi-parameter flow cytometry in whole blood. Tregs were identified as CD4^+^CD25^+^FoxP3^+^ cells. Monoclonal antibodies specific for surface markers (CD4, CD25; BD Biosciences, Franklin Lakes, NJ, USA) were added to 100 μL of whole blood and incubated for 20 min at room temperature (RT). Following incubation, red blood cells were lysed by adding 2 mL of 1× RBC lysis buffer (ThermoFisher, Waltham, MA, USA) and incubated for 10 min at RT. Intracellular FoxP3 expression was detected by using the FoxP3 staining buffer set (ThermoFisher) according to the manufacturer’s instructions. Flow cytometry data were collected by using the LSR Fortessa (BD Biosciences) and data were analyzed by using FlowJo v10 software (Tree Star, Ashland, OR, USA). The blood samples of each participant were measured once. Treg frequencies are presented as percentages of CD4^+^ T cells.

### 2.7. Cell Culture Experiments

Human naïve CD4^+^CD45RA^+^ T cells were isolated from buffy coats purchased from the Transfusion Medicine Department of the Medical University of Graz, Austria. First, peripheral blood mononuclear cells were isolated by density gradient centrifugation, and subsequently, CD45^+^CD45RA^+^ T cells were isolated by negative selection by using the MagniSort human CD4 naïve T cell enrichment kit (ThermoFisher).

Human CD4^+^CD25^+^FoxP3^+^ Treg cells were differentiated as previously described [39]. In brief, human naïve CD4^+^CD45RA^+^ T cells (1.5 × 10^6^ cells/mL) were cultured in serum-free X-vivo media (Lonza, Basel, Switzerland) supplemented with penicillin/streptomycin, Glutamax, 50 ng/mL IL-2 (Peprotech, London, UK) and 3 ng/mL TGF-β1 (R&D Systems, Minneapolis, Minn, USA) in the presence of immobilized anti-CD3 monoclonal antibodies (Biolegend). Where indicated polyphenols or the appropriate vehicle control were added. The polyphenols were used at previously tested non-toxic concentrations (Table S2). The polyphenols were elected as prominent ingredients of aronia juice [22] and obtained from Sigma Aldrich (ferulic acid Y0001013; rutin PHL89270; chlorogenic acid Y0000569; catechin PHL80992; vanillic acid 94770; syringic acid S6881, genitisic acid 85707; protocatechuic acid 03930590; salicylic acid S5922; 4-hydroxybenzoic acid 1609013) and were stored in powder form in the fridge at 4 °C. Working concentrations of the polyphenols were prepared by diluting in DMSO. Aliquots were treated with argon gas and stored at −20 °C in the dark.

Following 4 days of incubation, Treg percentages were measured by flow cytometry [40,41]. The experiments have been repeated three times in cells of different donors each and were measured once. The data is presented as mean ± standard deviation of the values of the three experiments.

### 2.8. Statistical Analysis

Statistical analysis was performed by applying the software SPSS Statistics version 27.0 (IBM, Armonk, NY, USA). According to the Shapiro–Wilk test, not all of the data was normally distributed. Thus, the data are presented as median (Md) and interquartile ranges (IQR). The Mann–Whitney U test was applied for group comparisons at the three time points of the investigation and the group comparison of delta that express the relative change between the investigations. The Kruskal–Wallis test was applied for the comparison of more than 2 groups and was followed by pairwise comparison in case the Kruskal–Wallis test was significant. For non-metric data, the chi-square and the Fisher’s exact tests were applied, respectively. For the assessment of the value progression over the course of the study the Wilcoxon rank-sum test and the Freidman’s test were applied, respectively. Any *p*-values below 0.05 were considered as significant and Bonferroni correction was applied for multiple testing.

To the best of our knowledge, no previous randomized controlled trial (RCT) aiming to find effects of nutritional interventions on the primary outcome Tregs was published at the conceptual phase of this study. Therefore, we could not estimate effect size. As a minimum number of 16 persons per group is needed to enable an effect of 1 standard deviation to be detected at 2p = 0.05 and 80% power, we chose *n* = 40 as with 20 participants per group sample size for this study. Other studies with similar interventions but other primary outcomes had sample sizes between 20 and 66 persons [28,42,43].

Statistical analysis of cell culture results was performed by using a two-tailed, unpaired t-test with Welch’s correction. The confidence level was 95% and results were considered significant with *p*-values lower than 0.05. The analysis of cell culture results was performed and the figures were created in GraphPad Prism version 9.0.0. (GraphPad Software, LLC. San Diego, CA, USA).

## 3. Results

### 3.1. Study Population

In total, 40 females were enrolled in the study of which 37 completed the intervention (first follow-up, V: 19; P: 18). A total of 35 participants (V:17, P:18) remained for the final investigation after the washout period. The participants of V and P were comparable in their main characteristics which are summarized in Table 1.

Anthropometric measures of the cohort were monitored throughout the study. The median (Md) of weight change from baseline (I) to the final investigation (III) within the whole cohort was 0.5 kg (Interquartile range (IQR): 1.6 kg) and the Md of the waist circumference change was 0.5 cm (IQR: 2.9 cm) from I to III. The weight and waist circumference changes between V and P did not differ significantly at all three measurement points.

Four participants of V and three participants of P reported smoking occasionally. The Fagerström test for assessing smoking behavior revealed an Md score of 0 (max. 4) in V and 0 (max. 2) in P, indicating no to low nicotine dependency.

### 3.2. Tolerability of the Study Drinks

A total of 94% of P and 47% of V tolerated the drinks well; however, 53% suffered from gastrointestinal complaints such as nausea and vomiting, diarrhea, stomach ache, cramps, and loss of appetite after aronia juice consumption. Thus, we additionally analyzed the data according to tolerability and separated the V into the verum group that tolerated the juice (Vt) and the verum group that reported on complaints after the juice consumption (Vc).

### 3.3. Nutritional Intake Data

The Md of the average total energy intake at baseline of V was 1974 kcal (IQR: 674 kcal) per day and 1895 kcal (IQR: 643 kcal) in P. The diet’s macronutrients contribution of total energy intake was similarly composed in both groups: 43% carbohydrates (IQR: 9%), 15% protein (IQR: 3%), 39% fat (IQR: 12%), and 1% alcohol (IQR: 3%) in V and 41% carbohydrates (IQR: 11%), 14% protein (IQR: 4%), 37% fat (IQR: 9%), and 2% alcohol (IQR: 5%). V consumed an Md of 23 g fiber (IQR: 17 g) and P an Md of 19 g fiber (IQR: 8 g).

Regarding the consumption of important polyphenol sources also the fruits and vegetable intake has been evaluated. The Md of fruits consumption was 133 g (IQR: 214 g) a day in V and 128 g (IQR: 178 g) in P, and the Md of the vegetable consumption was 202 g (IQR: 170 g) in V and 144 g (IQR: 111 g) in P. Dietary intakes did not change significantly over the study period. Important key nutrients at the baseline of the study are summarized in Table 1 and further nutritional information is provided in Supplemental Table S1.

### 3.4. Plasma Polyphenol Levels

**Figure 2:**
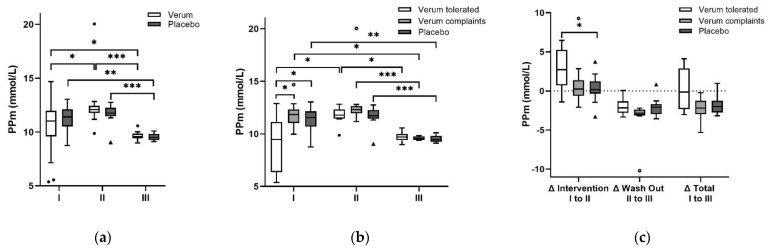
Progression of total plasma polyphenol microtiter (PPm) concentrations over the three investigation time points: (**a**) PPm increased significantly between baseline (I) and after the intervention (II) in the whole verum (V) group (*p* = 0.040); however, the significance did not remain after Bonferroni correction (*p* = 0.119) and PPm of the placebo (P) group did not change during the intervention. PPm decreased in both groups over time. (**b**) The separation of V into participants who tolerated the juice (Vt) compared to those who reported complaints (Vc) revealed significantly lower PPm in Vt at I (*p* = 0.010). PPm in Vt increased significantly from I to II (*p* = 0.024) whereas the PPm plasma concentrations of Vc and P remained constant. After Bonferroni correction, the increase in Vt did not remain significant. (**c**) The absolute difference of plasma PPm between the measurement time points (Δ) was the highest in Vt from I to II whereas PPm almost remained stable in Vc and P. The absolute increase in Vt was significantly higher compared to P (*p* = 0.015) and at least a trend towards significance was observed compared to Vc (*p* = 0.051). A decrease in PPm plasma concentrations was observed in all three groups during the washout phase. In Vt PPm almost returned to baseline concentrations after the washout period. *p*-values < 0.05 are marked with *, < 0.01 with **, and < 0.001 with ***. Outlayers of V and Vt are highlighted as black circles, of Vc as circles, and of P as black triangles. Abbreviations: V: verum, Vt: verum tolerated, Vc: Verum complaints, P: placebo, PPm: polyphenol microtiter, I: baseline, II: after intervention, III: after washout, Δ: difference between two measurement points.

Plasma PPm did not differ significantly between V and P at baseline (*p*= 0.495); however, when analyzed for tolerability groups plasma PPm was significantly lower in Vt compared to Vc (*p* = 0.010) and remained significant after Bonferroni correction (*p* = 0.031) and P (*p* = 0.019) which remained at least a trend after Bonferroni correction (*p* = 0.056). Plasma PPm increased significantly in Vt after the intervention with aronia juice from 9.47 (4.8) mmol/L to 11.8 (0.9) mmol/L (*p* = 0.024) but did neither change significantly in Vc (*p* = 0.480) nor in P (*p* = 0.230) from I to II. Plasma PPm decreased in the washout in all groups. The progression of plasma PPm concentrations of the groups and the differences in changes in PPm are depicted in Figure 2.

### 3.5. Oxidative Stress

Oxidative stress was assessed as the ratio between TAC and TOC and presented as oxidative stress index (OSI). The Md of OSI decreased significantly in V from 0.05 (0.13) AU to 0.02 (0.11) AU (*p* = 0.004) and increased again to the initial level of 0.05 (0.08) AU after the washout period (*p* = 0.001). Similar progress has been observed for P whose OSI decreased during the intervention from 0.05 (0.07) AU to 0.04 (0.06) AU (*p* = 0.030) and increased again during the washout period to 0.06 (0.16) AU (*p* = 0.001). Of note, the decrease in OSI in P after the intervention did not remain significant after Bonferroni correction (*p* = 0.091).

Regarding the tolerability groups, OSI did not differ significantly between the groups. However, the Md of OSI was the highest in Vc compared to Vt and P at baseline.

OSI decreased significantly in Vt (*p* = 0.024) and P (*p* = 0.040) from I to II; however, after Bonferroni correction, both did not remain significant. From II to III the OSI of all three groups increased significantly (Vt: *p* = 0.024, Vc: *p* = 0.018, P: *p* = 0.002) but only the increase in OSI in P remained significant after Bonferroni correction. The results of oxidative stress parameters are shown in Supplemental Figure S1.

### 3.6. Plasma Vitamin C Levels

The levels of plasma Vitamin C were almost the same at baseline in V with an Md of 16.6 (4.7) mg/L and in P with an Md of 16.7 (3.9) mg/L. Plasma Vitamin C changed only slightly throughout the study (n.s.). After the intervention, it increased by an Md of 1.4 (3.4) mg/L in V and remained the same in P with a change of −0.1 (3.35) mg/L in Md (n.s.). Despite these slight variations plasma vitamin C levels remained the same over the study. Additionally, within the tolerability groups, neither significant differences between the groups nor changes over the course of the study have been observed. The results of plasma vitamin C are shown in Supplemental Figure S2.

### 3.7. Regulatory T Cell (Treg) Frequencies

Treg frequencies did not differ significantly between V with an initial Md of 4.8 (3.1%) and P with an Md of 5.3 (2.6%). Tregs decreased in both groups over the course of the study. During the intervention, Tregs decreased continuously in V to 4.5 (3.7%) (*p* = 0.016) and during the washout period to 4.3 (1.7%) (*p* = 0.006) and both changes remained significant after Bonferroni correction. Despite a higher relative change of Tregs in P of −1.1 (2.1%) from I to II, the Friedman test was not significant (*p* = 0.070). The Wilcoxon rank-sum test would have revealed a significant decrease in P (*p* = 0.022) from I to II but no change from II to III or I to III was detected.

**Figure 3:**
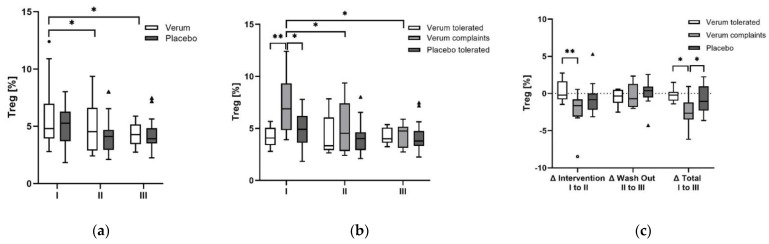
Progression of regulatory T-cell frequencies. (**a**) Treg frequencies decreased in V over the course of the study whilst Tregs in P remained almost constant. (**b**) When separated for tolerability groups Vc had significantly higher Treg % at baseline compared to Vt and P. Treg values decreased in Vc continuously during the study. (**c**) Tregs remained almost stable in Vt and P whilst a significant reduction in Treg % was observed for Vc. *p*-values < 0.05 are marked with *, < 0.01 with **. Outlayers of V and Vt are highlighted as black circles, of Vc as circles, and of P as black triangles. Abbreviations: V: verum, Vt: verum tolerated, Vc: Verum complaints, P: placebo, Tregs: regulatory T-cells, I: baseline, II: after intervention, III: after washout, Δ: difference between two measurement points.

Strikingly, when analyzed separately for tolerability groups, Vc had significantly higher Tregs initially compared to Vt (*p* = 0.005) and P (*p* = 0.017), whereas the significance of P turned to a trend (*p* = 0.051) after Bonferroni correction. After the intervention Tregs % decreased in Vc (*p* = 0.018) from I to III (*p* = 0.01), whereas the significance from I to II turned to a trend after Bonferroni correction (*p* = 0.055). In Vt and P, Tregs remained almost constant during the study (Figure 3). The Tregs frequencies of all three groups were within reported reference ranges [44].

### 3.8. Cell Culture Experiments

#### 3.8.1. In Vitro Differentiation of CD25^+^ FoxP3^+^ Tregs

**Figure 4:**
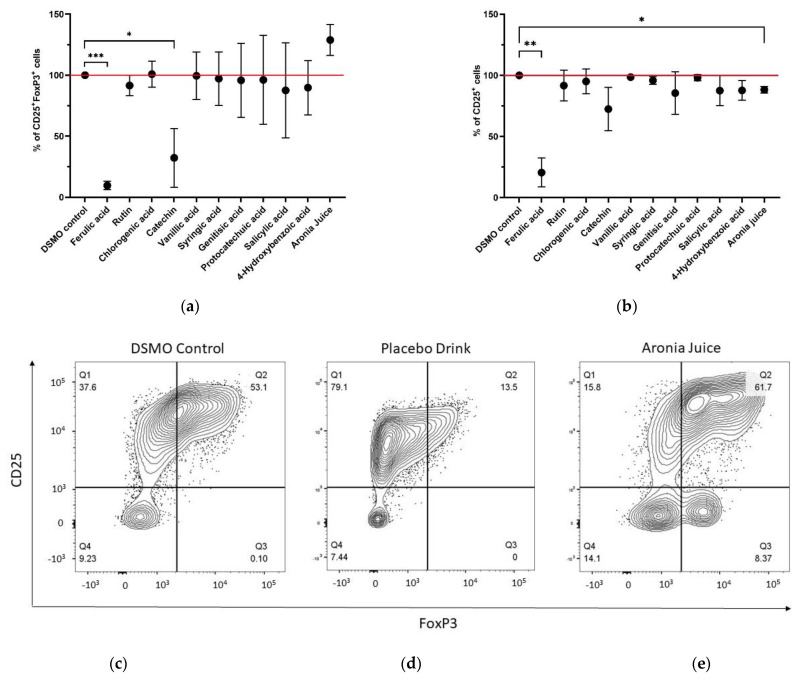
Results of the Treg differentiation experiments. The effects of polyphenols typically present in aronia juice and the natural aronia juice on Treg differentiation and T cell activation have been tested in vitro. (**a**) Ferulic acid (*p* = 0.0005) and catechin (*p* = 0.0393) showed significant inhibitory effects on Treg differentiation whereas the aronia juice slightly promoted Treg differentiation (n.s.); (**b**) Ferulic acid (*p* = 0.0072) and the aronia juice (*p* = 0.0163) significantly inhibited CD25 expression. (**c**–**e**) Representative flow cytometry plots from the Treg differentiation experiments. Following the treatment of naïve CD4^+^ T cells with IL-2 and TGF-β1, T cells were analyzed for the expression of CD4, CD25, and FoxP3. The representative plots show the percentages of CD25 and FoxP3 cells by gated CD4^+^ cells. Cultures treated with DSMO (**c**) and placebo drink (**d**) did not exhibit a CD25^-^FoxP3^+^ population; Cells treated with aronia juice (**e**) showed a unique population of CD25 negative cells that expressed FoxP3. *p*-values < 0.05 are marked with *, < 0.01 with **, and < 0.001 with ***. Abbreviations: Treg: regulatory T cell, iTreg: induced regulatory T cell, Ctrl: Control; DMSO: Dimethylsulfoxid.

The ability of the polyphenols contained in the aronia juice to influence the differentiation of human naïve CD4^+^CD45RA^+^ T cells into CD25^+^FoxP3^+^ Tregs was tested in an in vitro Treg differentiation assay. Cells treated with ferulic acid showed the strongest significant decrease in CD25^+^FoxP3^+^ Tregs (*p* = 0.005) with a mean reduction of 90.3% (±2.9%) of CD25^+^FoxP3^+^ Tregs compared to the DSMO control. Catechin also showed a significantly strong inhibitory effect (*p* = 0.0393) with a mean of 67.7% (±19.6%) reduced expression of CD25^+^FoxP3^+^ Tregs compared to the DSMO treated cells. The aronia juice had a promoting effect on CD25^+^FoxP3^+^ Treg differentiation with 28.8% (±10.3%) increased expression; however, this effect was not significant (*p* = 0.0586) (Figure 4a).

#### 3.8.2. Activation of T cells

The effect of the polyphenols on the activation of CD4^+^ T cells was evaluated by measuring the expression levels of the activation marker CD25. Thereby, ferulic acid inhibited T cell activation significantly by 79.5% (±9.6%) compared to DSMO control (*p* = 0.0072). Additionally, the aronia juice slightly but significantly decreased T cell activation (*p* = 0.0163) by reducing CD25 expression by 11.8% (±2.1%) on average. Moreover, catechin showed a tendency to decrease T cell activation by 27.6% ± 14.5%, but the results were not significant (*p* = 0.1148). Other polyphenols tested did not reveal significant effects on T cell activation (Figure 4b).

#### 3.8.3. Expression of a CD4^+^CD25^-^FoxP3^+^ T Cell Population following Treatment with the Aronia Juice

The addition of the aronia juice during the differentiation of naïve CD4^+^ T cells into Tregs resulted in a striking observation. The analysis of the cells by flow cytometry revealed the presence of a CD4^+^ FoxP3^+^ T cell population that however lacked the expression of CD25. This cell population was present only when the cells were exposed to the aronia juice and was not observed in the DMSO and placebo drink conditions (Figure 4c–e).

Representative flow cytometry plots for the polyphenols tested are shown in Supplemental Figure S3.

## 4. Discussion

In this study, we investigated the effects of aronia juice polyphenols in comparison to a polyphenol-free placebo drink on immunomodulation and oxidative stress in a cohort of healthy females. Additionally, we tested the effects of individual polyphenols and aronia juice on mechanisms of immunomodulation in cell culture experiments.

### 4.1. Immunomodulation and Oxidative Stress Response to the Intervention

We observed that around half of the participants did not tolerate the juice whereas the other half tolerated it well. Interestingly, we observed higher initial PPm and Treg levels in subjects who reported discomfort consuming 200 mL of aronia juice per day. Although the Tregs levels were higher in the complaints group, the values remained within normal reference ranges [44]. Tregs frequencies decreased during the intervention in this group whilst PPm increased. However, we cannot draw any conclusion from this observation with respect to the intervention since the initially elevated values could have had other unknown reasons. Plasma polyphenols increased in the verum group and showed the highest increase in Vt who tolerated the juice well. Thus, we assume the bioavailability of the aronia juice polyphenols.

Oxidative stress expressed as OSI decreased slightly in V, Vt, Vc, and P during the intervention and increased again to initial levels during the washout phase. This observation may be attributed to the vitamin and mineral content of both, the aronia juice and the placebo drink. Vitamin C plasma levels remained constant within the groups despite higher intakes through the intervention drinks. Interestingly, Vc had the highest initial OSI which in combination with the higher initial Treg levels may indicate unknown stressors before the study. However, both OSI and Tregs were within normal ranges of healthy individuals [38,44]. In total, the aronia juice had no striking effects on oxidative stress in our cohort. This is in line with the findings of Stankiewicz et.al 2021 [42] who investigated the effects of a 7-week intervention of 200 mL aronia juice per day in a group of young male football players and did not see any effect on oxidative stress. On the contrary, others found a positive impact of aronia juice on oxidative stress in vitro and in vivo [21].

### 4.2. Tolerability of Aronia Juice May Be Altered by Individual Factors

The concept of hormesis [45] may be of certain interest in the interpretation of the differences in tolerability of the aronia juice. A beneficial response to polyphenols may occur at low dose levels, whereas higher exposure may lead to adverse effects. Polyphenols serve as compounds of the natural defense system of plants and are toxic to their biological enemies. Thus, it has been proposed, that polyphenols may also act as stressors in vertebra cells at certain doses leading to the induction of the defense mechanisms. However, we hypothesize that the hormesis of polyphenols [11] may be dependent on individual factors. There may be a certain hormetic zone that supports the beneficial effects of polyphenols on the immune system. Since we observed divergent effects in half of our participants, this hormetic zone of aronia polyphenols is likely to be influenced by factors such as the gut microbiome composition [1] or genetic variations of bitter receptors that have been found along the whole gastrointestinal tract [46]. It has been proposed previously that the physiological benefit of polyphenols may differ according to the individuals` microbiome composition which may impact the bioavailability and functionality of polyphenols in the human organism on the one hand [1,19] and may be influenced by the individual dietary composition on the other hand [18,47].

### 4.3. Cell Culture Observation

#### 4.3.1. Immunomodulatory Effects of Individual Aronia Juice Polyphenols

To assess the effects of aronia juice polyphenols on immunomodulation mechanistically, in the first step individual polyphenolic compounds of the juice were investigated in cell culture experiments, using an induced regulatory T cell model (iTreg). Thereby, ferulic acid and catechin showed pronounced inhibitory effects on T cell activation and Treg differentiation.

In the case of ferulic acid, this may be attributed to the suppression of NF-κB signaling, being essential for Treg development, maintenance, and function [48]. A downregulation of NF-κB expression and lowering of the intensity of prostaglandin E2 as well as TNF-α by ferulic acid has been reported [49]. Additionally, it was previously shown that the treatment with ferulic acid ameliorated the inflammatory response induced by lipopolysaccharide (LPS) in bovine endometrial epithelial cells, shown by a decrease in proinflammatory cytokines such as IL-1β, IL-6, IL-8 and TNF-α after pretreatment with ferulic acid. Another anti-inflammatory aspect of ferulic acid is its ability to inhibit the degradation of IκB, the inhibitor of NF-κB, and to suppress the activation of MAPKs, which are signal transducers in inflammation response [50]. Ferulic acid and downregulation of NF-κB signaling were also seen in the decrease in the receptor activator of nuclear factor κB ligand (RANKL) mediated NF-κB activation, as shown in a study investigating the effects of ferulic acid in the context of bone erosion in rheumatoid arthritis [51]. In addition, its inhibitory effects on the expression of IL-1β, IL-6, and TNF-α via the modulation of NF-κB signaling, and ferulic acid have also been proven to attenuate the activation of the NLRP3 inflammasome (nucleotide-binding domain leucine-rich repeat and pyrin domain-containing receptor 3). The NLRP3 inflammasome is a protein complex, expressed in many cell types of the immune system such as monocytes, neutrophils, dendritic cells, and lymphocytes in the cytoplasm of the cells, acting as a pattern recognition receptor for inducing inflammatory response [52,53]. Ferulic acid was also reported to attenuate not only the expression of proinflammatory cytokines but also lipid peroxidation and lysosomal enzymes, with possible additional anti-oxidative properties. The same authors pointed out the binding of ferulic acid of NF-κB and NLRP3 proteins directly, which implies another potential mechanism of interaction [54]. Concerning the important modulation of TGF-β signaling by ferulic acid, Smad 4 as an important key player, is prevented from its nuclear translocation by ferulic acid and a decrease in phosphorylation of Smad 2/3 was observed [55]. TGF-β signaling is very important in the development of iTregs, as another possible mechanism of the inhibitory effect of ferulic acid [56].

Catechin and some of its derivatives have been postulated to interact with the DNA-binding site of NF-κB, which suggests inhibitory effects on NF-κB signaling as well [57]. Green tea catechins are also associated with an inhibition of NF-κB signaling [58] and suppression of proinflammatory cytokine expression and enzymes such as COX-2 or the inducible nitric oxide synthase (iNOS). Previously, not only the suppression of inflammatory mediators but also the enhancement of anti-inflammatory cytokine production including IL-4 and IL-10 were found [59].

Most dietary polyphenols are processed by the colon microflora. The main products reported in the literature are hydroxybenzoic acids and protocatechuic acid, which can originate from catechins, cinnamic acids such as ferulic acid, and many more [60]. Another study reported the detection of protocatechuic acid and other metabolites in plasma after consumption of aronia juice without any polyphenols that were initially present in the juice [61]. Therefore, it is important to investigate polyphenol in vivo effects more closely as non-fiber prebiotics and their possible immune reactions in the context of microbiota.

In conclusion, many polyphenols, but most significantly ferulic acid and catechin exerted inhibitory effects on both T cell activation and Treg development. The results we obtained indicated some anti-inflammatory properties of certain polyphenols that should be further investigated in the context of microbiota.

#### 4.3.2. Immunomodulatory Effects of Aronia Juice

The second step in assessing the immunomodulatory mechanisms of aronia juice polyphenols was to test the whole juice in comparison to DSMO control and the placebo drink to evaluate the impact of the food matrix and potential interaction of its nutritive components in the iTreg model. Thereby, the aronia juice revealed striking results in the iTreg assay. Interestingly, CD4^+^CD25^-^FoxP3^+^ cells emerged in response to aronia juice but not in any of the other conditions tested. Since the placebo drink had a comparable nutrient profile including macro- and micronutrients such as sugars, vitamins, and minerals, this observation may indicate the effects of the aronia polyphenols within the juice matrix. Noteworthy, in vivo the ingested juice is metabolized throughout various digestive processes which changes the bioaccessibility of polyphenols and extracts them from the original food matrix [62]. There are some approaches to predigest food for in vitro studies to mimic the digestive steps through the mouth, stomach, and the small and large intestine. However, it is known that the activity of bioactive compounds is different in in vitro and in vivo conditions and the predigestion models face certain limitations themselves [63]. Thus, we tested undigested juice in comparison to single polyphenols and the placebo drink as a model for the interactive effects of the food matrix and a polyphenol blend (Figure S3).

The CD4^+^CD25^-^FoxP3^+^ T-cell population has been observed in inflammatory diseases such as systemic lupus erythematosus [64,65] previously and was associated with rather adverse immune outcomes. However, for this CD4 subset, no consensus has been reached yet regarding its immunological functions [66].

Notably, we applied the juice at the maximum non-toxic concentration for the cells. This observation may support the hormesis hypothesis of polyphenols which suggests detrimental effects of too high doses of polyphenols [11,45]. Since aronia juice comprises multiple polyphenol classes, additive effects may contribute to pro-inflammatory effects at a certain level. However, this may be dependent on individual factors such as the microbial ability to process polyphenols and thus influences the bioaccessibility of polyphenols, and the individual tolerability response to the juice that may be associated with genetic variations for bitter receptor expression along the gastrointestinal tract [67,68,69].

We acknowledge limitations to this study. We included females only. Female cohorts have known variations in hormonal status over the course of the menstrual circle. Despite collecting information on the menstrual status of the participants, statistical analysis could not be adjusted for detailed menstrual circle information or hormonal variations. Since we investigated the participants within their usual lifestyle, habits such as smoking or alcohol consumption, stress, and other external factors may have influenced the outcome. Since the study groups were further divided into tolerability groups, the number of participants was small. The initially observed differences in the tolerability groups may have been attributed to other unknown factors. Thus, further studies need to investigate larger human cohorts to confirm our observations and to test the hypothesis drawn here. Regarding the methods applied, the analysis of total plasma polyphenols by a Folin–Ciocalteu approach may have interfered with other bioactives. The analysis of polyphenols by mass spectroscopy should be applied preferentially in further studies and despite TAC, the investigation of further antioxidant biomarkers (such as enzymes and endogenous compounds contributing to the redox system) may reveal a more detailed view of the individuals’ oxidant status. However, the strength of this study is the additional data from cell culture experiments, which revealed striking effects of the aronia juice and its polyphenols on immunomodulation.

## 5. Conclusions

Interestingly, we observed, that only half of the participants tolerated the administered aronia juice well. The tolerability was associated with lower initial plasma polyphenol and Treg frequencies, and the highest increase in plasma polyphenol concentrations. Oxidative stress did not change notably over the study. In cell culture, the polyphenols ferulic acid–a primary component of aronia juice and a degradation product of chlorogenic acid-and catechin had inhibiting effects on T cell activation and Treg differentiation and the aronia juice promoted the generation of a FoxP3^+^ cell population that lacked CD25. The effects of aronia juice polyphenols on immunomodulation may be dependent on individual factors. We propose that hormetic effects of polyphenols may be of certain interest in immunomodulation and hormesis may be influenced by individual factors such as gut microbiome composition and extraoral bitter receptors which may alter the tolerability of high polyphenols intake.

To conclude, we hypothesize that the differences in tolerability of the aronia juice may be attributed to hormesis effects. Thereby, the dose of aronia juice for optimized benefits may be dependent on intra-individual factors such as the microbial composition of the gut microbiome or the expression of bitter receptors along the gastrointestinal tract. Further analysis of the data derived from this study will include the gut microbiome composition, and metabolomics analysis to evaluate the metabolic turnover and outcome of the polyphenols.

## Figures and Tables

**Table 1:** Study population characteristics. Data are presented as median (IQR). *p*-values were derived from Mann–Whitney U test if not marked, from Fisher’s exact test if marked with ^†^, and from chi-square test if marked with ^† †^.

Study Population Characteristics	Verum	Placebo	*p*-Value
Number of participants (n)	20	20	
Age (years)	25 (7)	24 (5)	0.142
Smokers	4	3	1.000 ^†^
BMI (kg/m^2^)	21.2 (2.9)	21.6 (3.2)	0.841
Waist circumference (cm)	68.3 (9.3)	68.5 (4.8)	0.758
Drop out before first follow up	1	2	0.307 ^† †^
Drop out before second follow up	2	0	
**Study drink tolerability**			
Tolerated (n)	9	17	0.003 ^†^
Complaints (n)	10	1	
**Dietary intake at baseline**			
Energy (kcal/day)	1974 (674)	1895 (643)	0.547
Fruits (g/day)	133 (214)	128 (178)	0.277
Vegetables (g/day)	202 (170)	114 (111)	0.072
Polyphenols (mg/day)	626 (506)	522 (600)	0.779
Vitamin C (mg/day)	96 (86)	85 (35)	0.398
**Laboratory parameters at baseline**			
PPm (mmol/L)	11.0 (2.4)	11.4 (1.6)	0.495
OSI (AU)	0.051 (0.13)	0.048 (0.07)	0.925
Vitamin C (mg/L)	16.6 (4.7)	16.7 (3.9)	0.529
Vitamin C (µmol/L)	94.2 (26.4)	94.7 (22.3)	0.529
Treg (%)	4.8 (3.1)	5.3 (2.6)	0.738

## Data Availability

The raw data supporting the conclusions of this article will be made available by the authors, without undue reservation.

## Supplementary Materials

The following supporting information can be downloaded at: https://www.mdpi.com/article/10.3390/antiox11071283/s1, Figure S1: Progression of oxidative stress; Figure S2: Progression of Vitamin C plasma levels; Figure S3: Representative flow cytometry plots from the Treg differentiation experiments; Table S1: Nutrient profile of the study cohort at baseline; Table S2: Concentrations of polyphenols.
