# Supplementary material for: Immunomodulatory Effects of Aronia Juice Polyphenols—Results of a Randomized Placebo-Controlled Human Intervention Study and Cell Culture Experiments
Source: Antioxidants (Basel). 2022 Jun 28;11(7):1283. doi: 10.3390/antiox11071283 (PMC9312026; doi:10.3390/antiox11071283)
Supplement: Supplementary file 1 [file antioxidants-11-01283-s001.zip › antioxidants-1711953-supplementary.pdf]

Supplemental Data

Table S1. Nutrient profile of the study cohort at baseline. The data was derived from a 4-day food record prior the first blood draw and analyzed by the national specific nutritional software nut.s that is based on the German and Austrian food composition databases. No significant differences in nutrient profile was observed between the Verum and the Placebo group. Data are presented as median (IQR). P-values were derived from Mann-Whitney-U-test

Parameter	Verum	Placebo	p-value
Number of participants (n)	20	20	
**Nutrient profile of dietary intake at baseline**			
Energy (kcal/day)	1974 (674)	1895 (643)	0.547
**Macronutrients**			
Carbohydrates (g/day)	200 (76)	178 (78)	0.127
Sugar (g/day)	85 (41)	69 (35)	0.201
Protein (g/day)	69 (22)	64 (29)	0.398
Fat (g/day)	82 (37)	80 (29)	0.529
Saturated fat (g/day)	29 (17)	29 (13)	0.698
Omega-3-fatty acids (g/day)	1.6 (0.9)	1.4 (1.1)	0.461
Omega- 6-fatty acids (g/day)	13 (13)	14 (8)	0.947
Fiber (g/ day)	23 (17)	19 (8)	0.072
Alcohol (g/day)	2 (9)	5 (11)	0.620
**Elected Micronutrients with potential involvement in immune response**			
Retinol equivalents – Vitamin A (mg/day)	1.7 (1.8)	1.2 (0.7)	0.211
Vitamin D (μg/day)	2.3 (1.4)	1.7 (1.8)	0.052
Tocopherol equivalents- Vitamin E (mg/day)	16 (11)	15 (8)	0.602
Vitamin B6 (mg/day)	1.3 (0.8)	1.2 (0.5)	0.738
Vitamin B12 (μg/day)	3.4 (3.1)	3.6 (3.2)	0.883
Folate (μg/day)	241 (138)	204 (54)	0.091
Vitamin C (mg/day)	96 (86)	85 (35)	0.398
Iron (mg/day)	13 (5)	13 (5)	0.314
Zinc (mg/day)	11 (6)	9 (3)	0.461
Selenium (μg /day)	49 (19)	47 (24)	0.512
Copper (mg/day)	1.8 (1.2)	1.8 (0.4)	0.445
Magnesium (mg/day)	348 (220)	331 (113)	0.314

Table S2. Concentrations of polyphenols. The polyphenols tested in cell culture were previously tested for their maximum non-toxic concentrations in the cell culture model.

Polyphenol	Concentration [µg/ml]
Ferulic acid	500
Rutin	300
Chlorogenic acid	150
Catechin	125
Vanillic acid	250
Syringic acid	125
Genitsic Acid	125
Protocatechuic acid	62.5
Salicylic acid	125
4-Hydroxybenzoic acid	250

Oxidative Stress

Oxidative stress was assessed as the ratio between TAC and TOC and presented as oxidative stress index (OSI). OSI decreased in V after the intervention whereas it remained constant in P. OSI was lower in Vt compared to Vc at baseline. The most beneficial change of OSI was observed in Vc. OSI increased over the course of the wash out period in all groups. However, all changes observed were not significant (Figure S1).

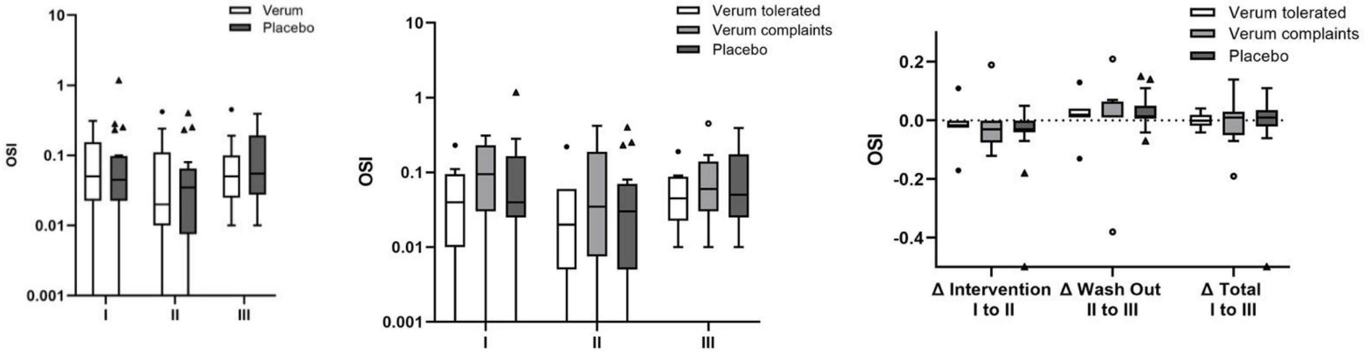

(**a**) (**b**) (**c**)

Figure S1. Progression of oxidative stress expressed as OSI as the ratio of TAC to TOC. (**a**) V and P had the same OSI at baseline. The Md of V decreased during the Intervention and increased again during the wash out phase whereas OSI remained constant in P. (**b**) within the tolerability groups Vt had lower OSI compared to Vc. OSI decreased in Vc during the intervention and was comparable with both other groups at the end of the study. **c**) OSI in Vc decresed slightly from I to II (n.s.) and got back to initial levels within the course of the washout phase. OSI levels of Vt and P remained almost constant over the study. p-values < 0.05 are marked with *, < 0.01 with **, and < 0.001 with ***. Outlayers of V and Vt are highlighted as black circles, of Vc as white circles, and of P as black triangles. Abbreviations: V: verum, Vt: verum tolerated, Vc: Verum complaints, P: placebo, OSI: oxidative stress index, TAC: total antioxidant capacity, TOC: total oxidative capacity, I: baseline, II: after intervention, III: after wash out, Δ: difference between two measurement points, Md: Median.

Plasma Vitamin C levels

The levels of plasma Vitamin C were almost the same at baseline in V and P and did only change slightly (n.s.) over the course of the study (Figure S2).

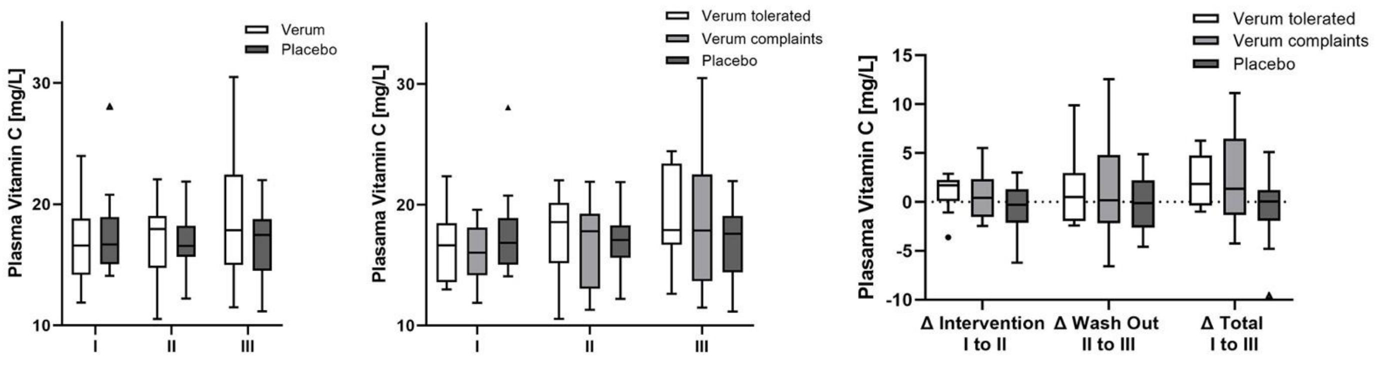

(**a**) (**b**) (**c**)

Figure S2. Progression of Vitamin C plasma levels. (**a**) Plasma Vitamin C levels increased slightly (n.s.) in V during the intervention whereas Vitamin C plasma levels remained constant in P. (**b**) The Md of Vitamin C had the highest increase in Vt from I to II . **c**) The absolute difference of Plasma Vitamin C changes over the course of the study is depicted. Plasma Vitamin C varied only slightly within the groups (n.s.). p-values < 0.05 are marked with *, < 0.01 with **, and < 0.001 with ***. Outlayers of V and Vt are highlighted as black circles, of Vc as circles, and of P as black triangles. Abbreviations: V: verum, Vt: verum tolerated, Vc: Verum complaints, P: placebo, I: baseline, II: after intervention, III: after wash out, Δ: difference between two measurement points, Md: Median.

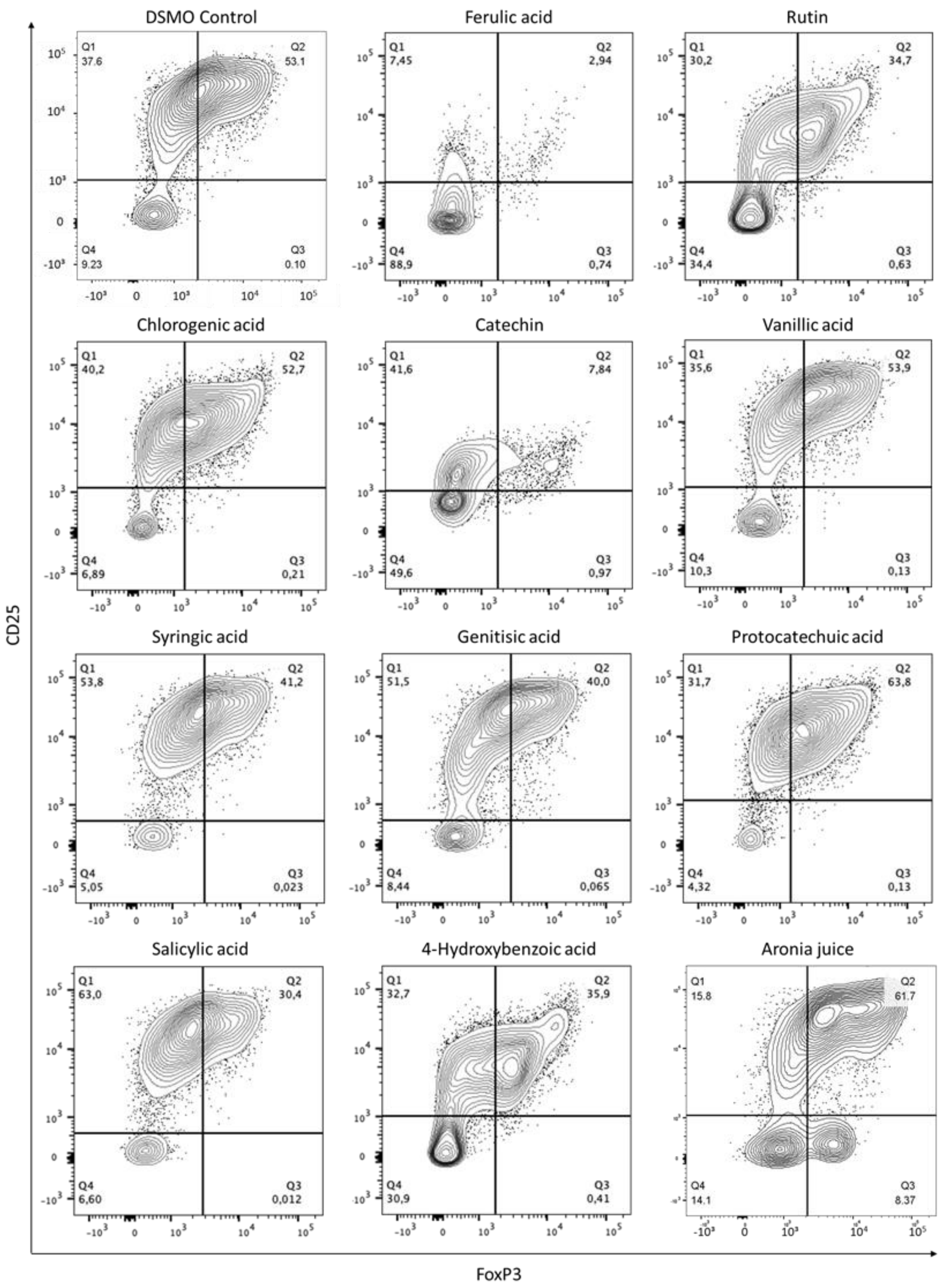

Figure S3. Representative flow cytometry plots from the Treg differentiation experiments. Following the treatment of naïve CD4⁺ T cells with IL-2 and TGF-β1, T cells were analyzed for the expression of CD4, CD25, and FoxP3. The representative plots show the percentages of CD25 and FoxP3 cells by gated CD4⁺ cells. Cultures treated with DSMO and the single polyphenols tested in this study. Only aronia juice treated cells showed a unique population of CD25 negative cells that expressed FoxP3.
